# Effects of weekly low-frequency rTMS on autonomic measures in children with autism spectrum disorder

**DOI:** 10.3389/fnhum.2014.00851

**Published:** 2014-10-21

**Authors:** Manuel Fernando Casanova, Marie K. Hensley, Estate M. Sokhadze, Ayman S. El-Baz, Yao Wang, Xiaoli Li, Lonnie Sears

**Affiliations:** ^1^Department of Psychiatry and Behavioral Sciences, University of LouisvilleLouisville, KY, USA; ^2^Department of Bioengineering, University of LouisvilleLouisville, KY, USA; ^3^College of Brain and Cognitive Neurosciences, Bejing Normal UniversityBejing, China; ^4^Department of Pediatrics, University of LouisvilleLouisville, KY, USA

**Keywords:** autism spectrum disorder, TMS, autonomic nervous system, electrocardiogram, skin conductance

## Abstract

The term autism spectrum disorder (ASD) describes a range of conditions characterized by impairments in social interactions, communication, and by restricted and repetitive behaviors. Autism spectrum disorder may also present with symptoms suggestive of autonomic nervous system (ANS) dysfunction. The objective of this study was to determine the effect of 18 sessions of low frequency (LF) repetitive transcranial magnetic stimulation (rTMS) on autonomic function in children with ASD by recording electrocardiogram (ECG) and electrodermal activity (EDA) pre- post- and during each rTMS session. The autonomic measures of interest in this study were R-R cardiointervals in EKG (R-R), time and frequency domain measures of heart rate variability (HRV) and skin conductance level (SCL). Heart rate variability measures such as R-R intervals, standard deviation of cardiac intervals, pNN50 (percentage of cardiointervals >50 ms different from preceding interval), power of high frequency (HF) and LF components of HRV spectrum, LF/HF ratio, were then derived from the recorded EKG. We expected that the course of 18 weekly inhibitory LF rTMS applied to the dorsolateral prefrontal cortex (DLPFC) would enhance autonomic balance by facilitating frontal inhibition of limbic activity thus resulting in decreased overall heart rate (HR), increased HRV (in a form of increased HF power), decreased LF power (resulting in decreased LF/HF ratio), and decreased SCL. Behavioral evaluations post-18 TMS showed decreased irritability, hyperactivity, stereotype behavior and compulsive behavior ratings while autonomic measures indicated a significant increase in cardiac interval variability and a decrease of tonic SCL. The results suggest that 18 sessions of LF rTMS in ASD results in increased cardiac vagal control and reduced sympathetic arousal.

## Introduction

Autism spectrum disorder (ASD) is characterized by difficulties in social interactions communication, and restricted and repetitive patterns of behaviors. In 2014, it was estimated by the Centers for Disease Control and Prevention (CDC) that ASD affects approximately 1 in 68 children (CDC’s Morbidity and Mortality Weekly Report, 2014). In addition to affecting neural development, it is also thought that ASD can manifest itself in abnormalities of autonomic nervous system (ANS) activity. Recent research suggests that some autistic individuals manifest an over-activation of the sympathetic branch of the ANS on a background of parasympathetic activity deficits (Ming et al., 2011). This bias creates an autonomic imbalance evidenced by a faster heart rate (HR) of little variability and increased tonic electrodermal activity (EDA; Zahn et al., 1987).

### Autonomic dysfunctions in autism

#### Heart rate variability

Several types of autonomic dysfunctions have been reported in autism, including increased basal sympathetic tone (Hirstein et al., 2001), as well as reduced baseline parasympathetic activity in association with increased baseline sympathetic tone (Toichi et al., 1999; Julu et al., 2001; Porges, 2001; Toichi and Kamio, 2003; Ming et al., 2004, 2005, 2011). Heart rate variability (HRV) measures are widely used in psychopathology research (Cohen et al., 2000; Thayer and Friedman, 2002) for assessment of phasic and tonic cardiac autonomic control (Berntson et al., 1997). Reduced HRV, specifically the attenuated power of high frequency (HF) component of the HRV (also called “respiratory sinus arrhythmia” [RSA]), is an indicator of limited psychophysiological flexibility (Berntson et al., 1997, 2008; Eckberg, 1997; Friedman and Thayer, 1998; Stein and Kleiger, 1999; Cohen et al., 2000). Several studies have shown that typical children show more HRV than autistic children (Hutt et al., 1975; Althaus et al., 1999; Jenkins et al., 2002), and that autistic children have unusually small deceleratory HR responses to stimuli (Palkovitz and Wiesenfeld, 1980; Coronoa et al., 1998; Porges, 2001). A recently published paper by Ming et al. (2011) reported evidence of reduced baseline parasympathetic activity and increased sympathetic tone in children with ASD. Another study by Bal et al. (2010) used RSA as a measure of cardiac vagal tone and compared RSA values between children with and without ASD. The study found that children with ASD had significantly lower RSA values and faster HR than those without ASD, which suggests decreased vagal cardiac regulation in autism. The clinical implications of chronic increased sympathetic activity and decreased vagal tone are poor control of HR and a tendency for tachycardia (Berntson et al., 1997, 2008). Therefore, analysis of the HRV, in particular the HF component of HRV along with other measures of heart beat variability (e.g., standard deviation of R-R intervals in electrocardiogram (ECG)) associated with parasympathetic activity, may provide important information regarding autonomic dysfunctions in autism.

Poor control of HR and vulnerability to tachycardia is an important consequence of chronic increased sympathetic activity and decreased vagal tone (Berntson et al., 1997, 2008; Coronoa et al., 1998; Friedman and Thayer, 1998). The baseline sympathetic over-arousal found in autism may reflect a condition of disinhibition, resulting from compromised baseline parasympathetic tone. Reduced fronto-limbic connectivity and poor prefrontal tonic inhibitory control over the limbic system (Loveland et al., 2008) might be one of the reasons for excessive excitation by the sympathetic branch of the ANS in ASD. Application of inhibitory rTMS to frontal cortex aimed at reducing the high cortical excitation/inhibition (E/I) ratio could be an effective technique for restoring normative fronto-limbic tonic inhibition, and for improving sympatho-vagal cardiac balance in autism.

#### Electrodermal activity

Studies of the ANS in autism have demonstrated several manifestations of abnormal sympathetic functions (Ming et al., 2004, 2005, 2011). Skin conductance response (SCR) studies in autistic children have shown a lack of the normal habituation in the magnitudes of SCR to the same stimulus over time (Udupa et al., 2007). Palkovitz and Wiesenfeld (1980) did not find differences in electrodermal reactivity to auditory stimulation compared to controls, but reported that the autistic group had a higher baseline skin conductance level (SCL). In addition, it has been reported that children with autism have a blunted autonomic arousal as indexed by SCL and SCR to visual or auditory social stimuli (Zahn et al., 1987; Hirstein et al., 2001; Ming et al., 2004, 2005, 2011). Angus (1970) found that children with ASD displayed more fluctuations in SCL compared to controls. Skin conductance response studies in autistic children have shown a lack of the normal SCR habituation to the same stimulus over time (Toichi and Kamio, 2003). Abnormal autonomic activity in during rest and during responses to stimulation in ASD was recently reported also in other studies (Benevides and Lane, 2013; Eilam-Stock et al., 2014). Furthermore, several of our own pilot studies also support excessive but less differentiated SCR to affective sounds, visual, and audio-visual stimuli in various affective stimulation tests (Sokhadze et al., 2012c; Dombroski et al., 2013) and positive changes following several experimental treatment approaches (Hensley et al., 2012, 2013; Sokhadze et al., 2012b; Dombroski et al., 2013). Since SCL is controlled solely by the sympathetic inputs (Williams et al., 2004; Boucsein, 2012), the above-mentioned effects are indicative of high sympathetic tone and low selectivity of sympathetic responses in autism.

### Neuromodulation approaches in treatment of autism

Recently there has been considerable interest on the effects of repetitive transcranial magnetic stimulation (rTMS) on cortical excitability. Biophysical foundations underlying TMS effects are reviewed in Wagner et al. (2009), while results of investigation of connectivity of the cortical structures during TMS using positron emission tomography (PET) was reported by Paus et al. (1997). Transcranial magnetic stimulation operates based on Faraday’s law of electromagnetic induction, which describes the process by which a changing magnetic field induces the flow of electric current in a nearby conductor, one preferentially standing at 90% to the magnetic field. Studies have indicated that low-frequency or “slow” rTMS (<1 Hz) increases inhibition of stimulated cortex, whereas high-frequency rTMS (>5 Hz) increases excitability of stimulated cortex. It has been proposed that the effect of slow rTMS arises from increases in the activation of inhibitory circuits (Pascual-Leone et al., 2000). We theorize that contrary to other inhibitory cells (i.e., basket and chandelier), whose projections keep no constant anatomical relation to the surface of the cortex, the geometrically exact orientation of double-bouquet cells and their location at the periphery of the minicolumn (the so-called inhibitory surround) makes them an appropriate candidate for induction by a magnetic field applied tangentially to the cortex. Over a course of treatment, slow rTMS may selectively depotentiate enhanced synaptic weights associated with pathological conditions, and, in the case of ASD, may lower the ratio of cortical excitation to cortical inhibition. Safety of TMS application in children were reviewed in several reports (Quintana, 2005; Garvey and Mall, 2008).

Transcranial magnetic stimulation has already shown to be an effective neuromodulatory tool capable of altering ANS functions. In a paper by Udupa et al. (2007) researchers compared rTMS with antidepressant therapy to address the autonomic imbalance associated with depression. The authors found that rTMS not only produced antidepressant effects, but also “corrected” the autonomic balance. The researchers used HRV measures as evidence that rTMS did in fact reduce the sympathetic-to-parasympathetic ratio thus improving the sympatho-vagal balance. In our previous studies slow rTMS was shown to improve both evoked EEG gamma activity and error processing in individuals with ASD. Baruth et al. (2010a) compared evoked gamma activity in the early stages of visual processing between individuals with ASD and neurotypicals using Kanizsa illusory figures in a visual oddball task. In autistic individuals, evoked gamma activity was not discriminative of stimulus type, whereas control subjects displayed early gamma-power differences between target and non-target stimuli (for a review of gamma activity see Casanova et al., 2013). Individuals with ASD underwent 12 sessions of rTMS and repeated the Kanizsa test. Results showed improvement in discriminatory gamma activity between target and non-target stimuli, as well as improvement in responses on behavioral questionnaires. In a study by Sokhadze et al. (2012a) TMS was used to improve error processing in children with ASD, as measured by event-related potentials (ERP) associated with response to errors, such as error-related negativity (ERN). Post-TMS results showed significant differences in the response-locked ERPs such as ERN, as well as behavioral response monitoring measures indicative of improved error monitoring and correction function (Sokhadze et al., 2012a). In another pilot study we reported minute-by-minute changes of HR, HRV indices, and SCL during 12 session of rTMS in children with autism (Hensley et al., 2012, 2013). In particular, we noted a decrease in the LF component of HRV and a decrease of SCL during 10 min of rTMS session indicative of decreased sympathetic activity.

The dorsolateral prefrontal cortex (DLPFC) was selected as a target for stimulation in our rTMS studies based on the topographical analysis of minicolumnar morphometry in cortices varying in cytoarchitectural differentiation: paralimbic, high-order (heteromodal) association, modality specific (unimodal) association, and idiotypic areas (Casanova et al., 2006). Neuroanatomical studies indicated that minicolumnar abnormalities in autism occur in a gradient that parallels connectivity; high-order association areas exhibiting salient abnormalities while idiotypic areas apparently being sparred. In addition, several of our recent publications have demonstrated positive behavioral, clinical and electrophysiological functional outcomes of rTMS when stimulating the DLPFC in children with autism (Sokhadze et al., 2009a,b, 2010a,b, 2012a; Baruth et al., 2010a,b, 2011; Casanova et al., 2012).

It is doubtful whether a pervasive neurodevelopmental disorder such as ASD could be explained in terms of pathology within a single brain area, i.e., DLPC. However, “normalizing” an area like the DLPFC whose physiology depends on distributed networks may provide beneficial cascading effects at secondary sites (Walsh and Pascual-Leone, 2003). Due to the anatomical and functional connectivity of the DLPC, we expected the TMS-based intervention not to be limited to the site of magnetic stimulation but rather to generalize to other cortical and subcortical areas. In effect results of our pilot studies (Sokhadze et al., 2009a,b, 2010a, 2012a) have shown changes of ERP and induced electroencephalographic (EEG) gamma oscillations not only in the frontal lobe but also in distal cortical areas (parietal, parieto-occipital, etc.). Effects of rTMS over DLPFC are possibly extended to paralimbic and limbic structures as well and may manifest themselves in ANS activity changes.

We hypothesized that rTMS stimulation applied bilaterally to the DLPFC would improve autonomic measures, more specifically, it was predicted that it would lower sympathetic arousal and normalize autonomic balance. Heart rate variability and SCL measurements were used to track changes in autonomic balance caused by rTMS. We chose to use HRV and SCL as indicators of the effectiveness of rTMS treatment because they are largely controlled by the ANS. The first measure, HRV, allowed us to observe differences in cardiac autonomic control, while the second measure, SCL, is controlled solely by sympathetic inputs and is therefore an excellent indicator of sympathetic nervous system activity. The expected outcomes were an increase in average R-R intervals in ECG, an increase in standard deviation of R-R intervals, an increase in the HF component of HRV, a decrease in the LF component of HRV, a decrease in the LF/HF ratio, increase in pNN50, as well as a decrease of SCL. We also predicted that the proposed intervention would provide for improvements in irritability, hyperactivity and repetitive behavior rating scales on the Aberrant Behavior Checklist (ABC; Aman and Singh, 1994) and Repetitive Behavior Scale (RBS; Bodfish et al., 1999). This is a proof of concept study aimed at defining the putative existence of positive effects as well as the effect size of our TMS intervention in a population of ASD individuals. It is hoped that the study will establish the potential to pursue future trials of adequate sample size using a sham control population. In this regard the present study does not constitute a clinical trial.

## Methods

### Subjects

In this study, we investigated the activity of the ANS during rTMS treatment in 18 children with ASD (14 boys and 4 girls, mean age 13.1 years, SD = 2.2). Participants with ASD were recruited through the University of Louisville Weisskopf Child Evaluation Center (WCEC). Diagnosis was made according to the DSM-IV-TR and further ascertained with the Autism Diagnostic Interview-Revised (ADI-R; Le Couteur et al., 2003) by Dr. Sears, who also did pre- and post-TMS clinical evaluations. All participants were high-functioning children with ASD and with full-scale IQs >80 assessed using the Wechsler Intelligence Scale for Children, Fourth Edition (WISC-IV; Wechsler, 2004). Participating subjects and their parents (or legal guardians) were provided with all information regarding the study, and the consent and assent forms approved by the IRB were reviewed and signed. Sixteen ASD subjects out of 18 enrolled in the study completed all 18 sessions of rTMS. Two subjects (both boys) completed only 14 sessions and dropped out of study due to family circumstances. Therefore, our retention rate in the study was 88.8%. Two subjects (one boy, one girl) were excluded from data analysis because they were active junior track-and-field athletes (long distance runners) and their cardiac activity was affected by the changes in their intense physical exercise regimen.

The study complied with all relevant national regulations and institutional policies and has been approved by the local Institutional Review Board (IRB). Participating subjects and their parents (or legal guardians) were provided with full information about the study including the purpose, requirements, responsibilities, reimbursement, risks, benefits, alternatives, and role of the local IRB. The consent and assent forms approved by the IRB were reviewed and explained to all subjects who expressed interest to participate. All questions were answered before consent signature was requested. If the individual agreed to participate, both she/he and parent/guardian signed and dated the consent or assent form and received a copy countersigned by the investigator who obtained consent.

### Low frequency repetitive TMS procedure

A trained electrophysiologist delivered rTMS using a Magstim Rapid 220 system (Magstim Co, Whitland, UK). Patients were seated in a leather chair and fitted with a swimming head cap. Motor threshold (MT) was determined in the following manner: mild supra-threshold stimulations was administered over the left motor cortex to determine the optimal area for stimulation of the *abductor pollicis brevis* (APB) muscle. The output of the machine was increased by 7% each time until the least amount of machine power that induces a 50 µV deflection or a visible twitch is identified in four out of five trials over the cortical area controlling the contralateral APB. Surface electrodes were attached over the APB and *first dorsal interossi (FDI)* areas. Electromyographic (EMG) responses (motor evoked potentials) were recorded using the C2 J&J Engineering Inc. (Poulsbo, WA) physiological data acquisition system interfaced with Magstim TMS device. Similar procedure was applied to determine MT for the right hemisphere. The TMS treatment course was administered once per week for 18 weeks over the DLPFC (six over the left, six over right, and six equally over the both left and right hemispheres). The site for stimulation was placed 5 cm anterior to, and in a parasagital plane to the site of maximal APB stimulation. The figure-eight coil, with a 70-mm wing diameter was kept flat over the scalp. Stimulation was performed at 0.5 Hz and 90% of resting MT, with a total of 160 pulses/per day (session had 8 trains by 20 pulses, with a 20-s interval between the trains, for additional procedure detail see Casanova et al., 2012; Sokhadze et al., 2012a).

### Autonomic monitoring procedure

#### Physiological monitoring

For 3–5 min before rTMS, during ~10–12 min rTMS session, and immediately after the completion of the TMS for another 3–5 min the subjects had their physiological activity monitored and recorded. Therefore, all autonomic measures were recorded during each rTMS session in every participant for several minutes preceding TMS administration, then during TMS procedure, and also for several minutes after TMS session. For data analysis in this particular study were included only data during administration of TMS. We used approximately 10 min long period to calculate HRV variability measures (RR intervals, SDRR, LF and HF of HRV) derived from an artifact free ECG recording and mean SCL. In our other pilot studies (Hensley et al., 2012, 2013) in addition to analysis of mean values of autonomic measures it was analyzed as well minute-by-minute values of HR and SCL.

**Figure 1:**
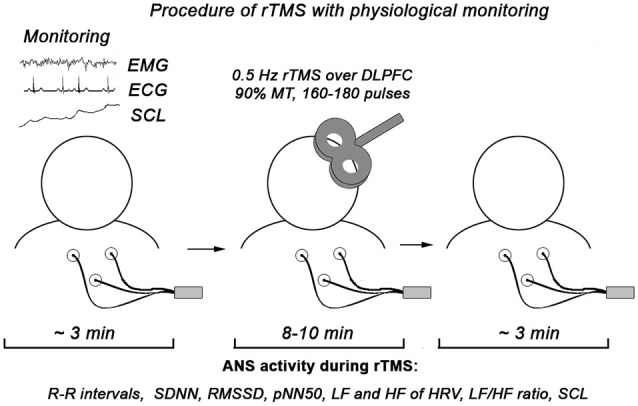
**Procedure of physiological monitoring during rTMS session**. In the statistical analysis we used only autonomic data recorded during rTMS (approximately 10 min).

The monitoring of ANS activity was conducted using C2 J&J Engineering Inc. (Poulsbo, WA) device with specialist USE-3 software application. The procedure of autonomic monitoring includes presentation of HRV measures in a form of cascading HRV spectrum, individual HRV components and SCL (both tonic and phasic changes) with visual and auditory feedback for experimenter. All physiological measures were analyzed both on- and off-line. Schematic presentation of the procedure is depicted at the Figure 1.

#### Measurement of the ANS dependent variables

Electrocardiogram, electromyogram (EMG), pneumogram (PNG), and EDA were acquired (1024 Hz sampling rate for EMG and ECG, 128 Hz for PNG and EDA) by a C-2 J&J Engineering Inc. physiological monitoring system with USE-3 software (Physiodata, Poulsbo, WA). Three Ag/AgCl electrodes (El-503, Biopac Systems, Inc., CA) were attached for measurement of Lead II ECG, 3 Ag/AgCl electrodes (EL-501 from Biopac) for EMG recording from the right hand, and PNG was recorded with a strain gauge transducer. Electrodermal activity was recorded by Ag/AgCl electrodes (EL-507 by Biopac with Unibase isotonic gel) attached to the distal phalanx of index and middle fingers to measure SCL.

##### Cardiovascular activity

Average R-R intervals in ECG (R-R), standard deviation of all normal R-R (NN) intervals (SDNN), Square root of the mean of the squares of successive NN interval differences (or the average change in interval between beats)— RMSSD, the percentage of intervals >50 ms different from preceding interval (pNN50); frequency domain HRV measures such as power of HF, LF, very low frequency (VLF) components, and the ratio of the LF over the HF (LF/HF ratio is used as an indirect autonomic balance index) of HRV are calculated as time domain and frequency domain cardiac activity measures (Kleiger et al., 2005). Artifact-corrected at least 5 min long recording epochs were analyzed with Fast Fourier Transformation (FFT) to assess HRV. Integrals of the spectrum in 0.04–0.15 Hz (LF of HRV) and 0.15–0.40 Hz (HF of HRV) bands were measured (in ms^2^). All HRV data was analyzed off-line using Kubios HRV software v. 2.0 (University of Kuopio, Finland). Heart rate variability interpretation was following concepts: (1) The HF component of HRV is often referred to as RSA and is assumed to be the non-invasive index of parasympathetic influences on the heart (Berntson et al., 1997; Sohn et al., 2001); (2) the LF component of HRV has been linked to sympathetic nervous system activity and sympatho-vagal balance by numerous studies (Pagani et al., 1986; Malliani et al., 1994). Other studies have shown that the LF variability is rather a reflection of both sympathetic and vagal influences related to baroreflex mechanisms (Berntson et al., 1997). It is thought that changes in blood pressure amplitude may cause a vagally-mediated baroreflex responses as well as changes in LF variability.

##### Respiratory activity

Respiration rate on per minute basis and peak respiration frequency were calculated. These measures were used to control HF peak in HRV related to respiratory frequencies in HRV and were not used as dependent measures.

##### Electrodermal activity

Skin conductance level (in µS) and amplitude of the SCR, defined as fluctuation with more than 0.02 µS increment (Boucsein, 2012), NS.SCR—number of non-specific SCR (per min) were calculated, but only SCL was used as dependent variable in this study. The main reason of excluding NS.SCR measure from analysis was related to the consideration that some of the SCR might reflect auditory stimulation response to clicks produced by the TMS coil and could be considered as non-specific SCRs.

### Behavioral outcomes

For the evaluation of social and behavioral functioning we utilized caregiver reports and clinician ratings of improvement. Every participant was evaluated before TMS course and within 2 weeks following TMS treatment. Aberrant Behavior Checklist (Aman and Singh, 1994; Aman, 2004) is a clinician administered rating scale to assess Irritability, Lethargy/Social Withdrawal, Stereotypy, Hyperactivity, and Inappropriate Speech based on parent/caregiver report. *Social Responsiveness Scale (SRS)*.*Repetitive Behavior Scale-Revised* (*RBS-R*, Bodfish et al., 1999) is a caregiver completed rating scale assessing stereotyped, self-injurious, compulsive, ritualistic, sameness, and restricted range (Bodfish et al., 1999).

### Statistical analysis

The primary statistical analyses included linear regression estimation of each autonomic dependent variable over 18 sessions of rTMS course, paired sample *t*-test of mean values of dependent ANS variables at the first and last session of the rTMS course, and paired sample *t*-test of pre-TMS and post-TMS behavioral measures. For each dependent autonomic variable analyzed using *t*-test, normality of distribution was analyzed to ensure appropriateness for the test, and 95% confidence intervals (95% CI) were included in outcome.

## Results

### Autonomic activity measures

#### Time-domain measures of HRV (R-R intervals, SDNN, RMSSD, pNN50)

**Figure 2:**
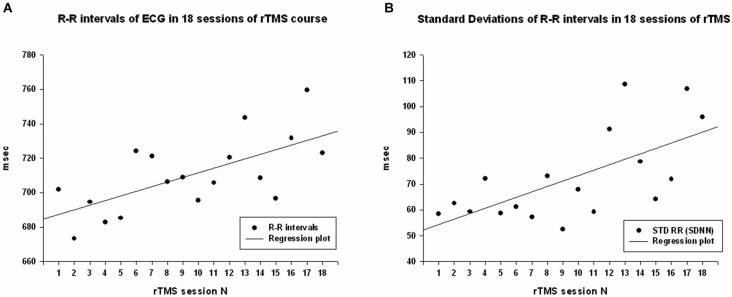
**(A)** Scattergram of linear regression of R-R intervals in ECG across 18 sessions of rTMS in 14 children with ASD. The mean values of R-R intervals show linear increase over the rTMS course. **(B)** Scattergram of linear regression of standard deviations of R-R intervals (SDNN) across 18 sessions of rTMS course shows linear increase.

Cardiointervals in ECG (R-R intervals) showed a statistically significant linear regression over 18 sessions of rTMS (*R* = 0.661, *R*^2^ = 0.437, *y* = 2.69*x* + 684.5 ms, *t* = 3.52, *p* = 0.003, observed power = 0.868 at *α* = 0.05, Figure 2A). *T*-test showed that R-R intervals increased statistically from the first to the last rTMS session (from 684.7 ± 90.9 ms to 723.8 ± 96.5 ms, mean increase being 39.08 ± 53.6 ms, 95% CI from 70.04 to 8.13 ms, *t*_(13)_ = 2.72, *p* = 0.017). Standard Deviations of R-R intervals showed statistically significant linear increase over 18 sessions of rTMS (*R* = 0.645, *R*^2^ = 0.417, *y* = 2.09*x* + 52.2 ms, *t* = 3.38, *p* = 0.004, observed power = 0.844 at *α* = 0.05, Figure 2B) and *t*-test showed that SDNN increased statistically from the first to the last rTMS session (from 60.6 ± 20.4 ms to 99.7 ± 74.7 ms, mean increase being 39.09 ± 66.7 ms, 95% CI from 77.6 to 0.53 ms, *t*_(13)_ = 2.19, *p* = 0.047). Increase of the RMSSD was only marginally linear (*R* = 0.473, *R*^2^ = 0.224, *y* = 1.48*x* + 52.8, *t* = 2.15, *p* = 0.047, observed power = 0.512 at *α* = 0.05, i.e., below the desired power of 0.800). Changes in pNN50 both across 18 sessions and between the first and last session of rTMS did not reach significance level (both >0.05).

#### Frequency-domain measures of HRV (LF and HF of HRV, LF/HF ratio index)

**Figure 3:**
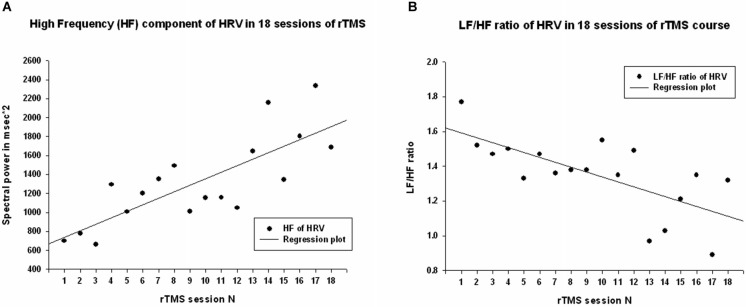
**(A)** Scattergram of linear regression of HF component of HRV across 18 sessions of rTMS in 14 children with ASD. The mean values of HF component of HRV show linear increase over the rTMS course. **(B)** Scattergram of linear regression of LF/HF ratio (cardiac autonomic balance index) across 18 sessions of rTMS course shows linear decrease.

**Figure 4:**
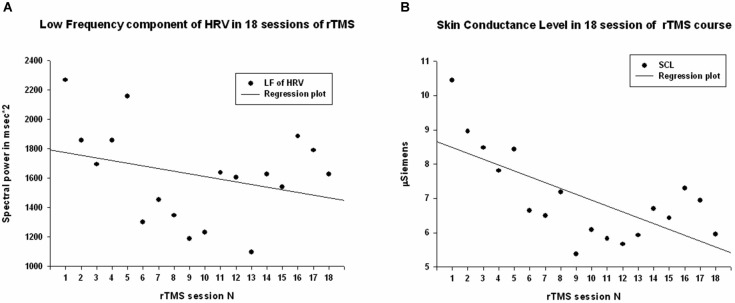
**(A)** Scattergram of linear regression of LF component of HRV across 18 sessions of rTMS in 14 children with ASD. The mean values of LF show tendency to decrease over the rTMS course but the trend was not reaching significance level. **(B)** Scattergram of linear regression of skin conductance level (SCL) across 18 sessions of rTMS course shows significant linear decrease.

Power of HF component of HRV showed a strong statistically significant linear increase (*R* = 0.788, *R*^2^ = 0.621, *y* = 68.6*x* + 671.9 ms^2^, *t*_(18)_ = 5.12, *p* < 0.001, observed power = 0.985 at *α* = 0.05 Figure 3A), *t*-test did show statistical increase (by 1249 ± 1556 ms^2^, 95% CI from 2147 to 350 ms, *t*_(13)_ = 3.00, *p* = 0.01). The Power of LF component of HRV showed a tendency towards linear regression but was not statistically significant (*R* = 0.247, *y* = −15.23*x* + 1775.4 ms^2^, *t* = −1.02, *p* = 0.323, observed power = 0.163 at *α* = 0.05, not significant, well below the desired power of 0.800. Figure 4A), *t*-test also did not show statistical difference (*p* > 0.05). The LF/HF ratio index (linear regression shown at Figure 3B) did show statistically significant linear decrease, *R* = 0.691, *R*^2^ = 0.478 *y* = −0.28*x* + 1.619, *t* = −3.83, observed power = 0.913 at *α* = 0.05) and also decreased significantly from the first to last rTMS session (by 0.48 ± 0.81, 95% CI from 7.64 to 2.89, *t*_(13)_ = 2.23, *p* = 0.044).

#### Skin conductance level

Skin conductance level showed statistically significant liner regression over 18 sessions of rTMS (*R* = 0.681, *R*^2^ = 0.464, *y* = −17*x* + 8.65, *t* = −3.71, *p* > 0.002, observed power = 0.948 at *α* = 0.05, Figure 4B), and *t*-test yielded statistically significant decrease from the first to the last rTMS session (from 10.22 ± 4.53 to 5.84 ± 3.41 µS, mean decrease −4.37 ± 5.65 µS, *t*_(13)_ = 2.89, *p* = 0.013).

### Behavioral evaluations post- TMS

**Figure 5:**
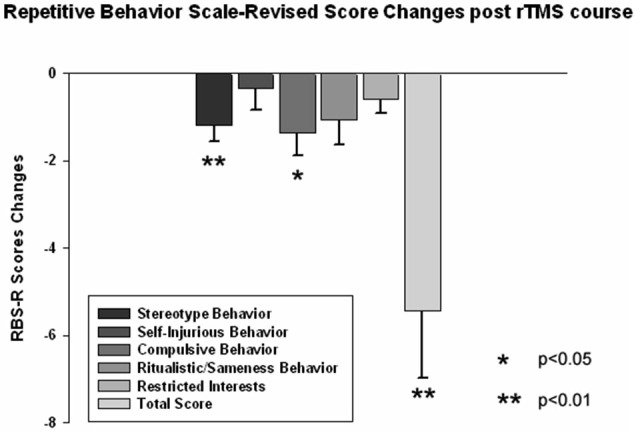
**Changes of Repetitive Behavior Scale (RBS-R) scores post-TMS as compared to baseline levels in children with ASD (*N* = 14)**. Stereotype Behavior, Ritualistic Behavior and Total RBS scores decreased significantly.

**Figure 6:**
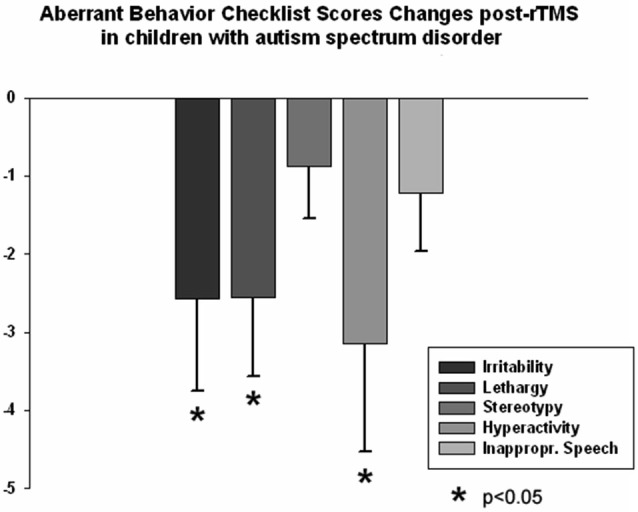
**Changes of Aberrant Behavior Checklist (ABC) scores post-TMS as compared to baseline levels in children with ASD (*N* = 14)**. Irritability, Lethargy, and Hyperactivity rating scores decreased significantly post-TMS.

The ABC and RBS behavioral checklists showed significant improvements in several areas. We found a significant decrease in stereotype repetitive and restricted behavior patterns following 18 sessions of bilateral rTMS as measured by the RBS-R (Bodfish et al., 1999) when analyzed using a paired sample Student’s *t*-test. Total RBS-R score decreased from 25.4 ± 14.0 to 19.8 ± 10.9, with the mean decrease being −5.44 ± 6.49, *t*_(13)_ = 3.55, *p* = 0.002. Changes in individual subscale rating scores are shown in Figure 5, where Stereotypic Behavior Subscale shows significant decrease (from 5.94 ± 4.30 to 4.76 ± 3.84, mean change −1.17 ± 1.59, *t*_(13)_ = 3.05, *p* = 0.008) and Ritualistic/Sameness Behavior Subscale scores show a significant decrease (−1.35 ± 2.02, *t*_(13)_ = 2.52, *p* = 0.022). We also found a significant reduction in Irritability subscale as measured by the ABC (from 10.53 ± 6.86 to 7.95 ± 5.56, mean change −2.57 ± 5.17, *t*_(13)_ = 2.17, *p* = 0.044). Lethargy subscale of the ABC showed a similar score reduction (−2.55 ± 4.32, *t*_(13)_ = 2.50, *p* = 0.023) while Hyperactivity showed an even greater reduction (from 13.53 ± 10.91 to 10.37 ± 9.36, −3.15 ± 6.08, *t*_(13)_ = 2.27, *p* = 0.035). Changes of individual subscale rating scores are depicted at the Figure 6.

### Overview of results

Results of the HRV analysis show several measures with significant differences between pre- and post-TMS therapy. Table 1 below shows the *t*-test results for RR interval, SDNN, HF component of HRV, LF/HF ratio, and SCL. Table 1 shows that there was a significant increase in the R-R interval from pre- to post-TMS treatment. This result can also be interpreted as a decrease in HR since the R-R interval is the time between successive heartbeats. The *t*-test also reveals significant increases in SDNN and HF power, as well as significant decreases in the LF/HF ratio and SCL.

**Table 1:** **Regression equations and statistics of linear regression of autonomic dependent variables over the 18 session long rTMS course in 14 children with ASD**.

Measure	Units	*t*	*p*-value	*R*	R^2^	Regression equation	Power at *α* = 0.05
**RR**	ms	3.52	0.003	0.661	0.437	*y* = 2.696*x* + 684.57	0.868
**SDNN**	ms	3.38	0.004	0.645	0.417	*y* = 2.098*x* + 52.28	0.844
**RMSSD**	ms^2^	2.15	0.047	0.473	0.224	*y* = 1.480*x* + 52.80	0.512
**LF power**	ms^2^	−1.02	0.323	0.247	0.061	*y* = −15.23*x* + 1775.4	0.163
**HF power**	ms^2^	5.12	<0.001	0.788	0.621	*y* = 68.65*x* + 671.9	0.985
**LF/HF ratio**	N/A	−3.83	0.001	0.691	0.478	*y* = −0.028*x* + 1.619	0.913
**SCL**	µS	−3.71	0.002	0.681	0.464	*y* = −0.17*x* + 8.65	0.948

Regression analysis was completed to observe trends during the entire 18 session TMS course. Table 2 shows the results of regression analysis. Regression analysis shows that the trend in each measure was significant for all analyzed, expect for LF power. Although there was a negative trend for LF power observed over the 18 sessions of TMS, it did not reach significance. NN50 (count of R-R intervals differing by >50 ms from the preceding interval) does show a significant positive trend; however, because the length of each physiological recording session was not uniform, regression analysis results of NN50 are not a reliable reflection of this measure. pNN50, the percent of RR intervals differing by >50 ms from the preceding interval, did not show a significant positive trend. Figures 2–4 show the regression analysis for individual measures. Behavioral questionnaires also demonstrated significant improvements both for the ABC and RBS rating scales (Figures 5, 6).

**Table 2:** **Changes of dependent variables of autonomic activity from the first to the last session of rTMS treatment course in 14 children with ASD**.

Pairs			Paired differences									
	Units	Mean	Std. Dev.	95% CI		*t*	df	*p*-value
				Lower	Upper			
**RR** post-pre	ms	39.08	53.61	70.04	8.13	2.73	13	0.017
**SDRR** post-pre	ms	39.09	66.78	77.65	0.54	2.19	13	0.047
**HF** post-pre	ms^2^	1249.3	1556.1	2147.8	350.8	3.00	13	0.010
**LF/HF** post-pre	N/A	−0.48	0.81	−0.01	−0.95	−2.23	13	0.044
**SCL** post-pre	µS	−4.37	5.65	−1.11	−7.64	−2.89	13	0.013

## Discussion

All novel studies involving neuromodulation techniques in children should proceed with caution. Transcranial magnetic stimulation is a non-invasive intervention, which could be a potential strategy for early intervention for autism; however, the dose, duration, and type of rTMS stimulation for such intervention in children as well as effects on vital functions need to be carefully investigated and documented. This need requires proof of concept studies when testing the feasibility of using rTMS in order to modulate autonomic activity in ASD.

Results of our study indicate that HRV and EDA are noninvasive and effective ways of gathering information about ANS functioning during rTMS therapy in autism. Accelerated HR in association with lower HRV indexed by high LF/HF ratio and low SDNN along with high electrodermal activity (SCL) found in children with ASD at the pre-treatment stage are indicators of excessive sympathetic and reduced parasympathetic activation in ASD resulting in limited psychophysiological flexibility and behavioral rigidity. We investigated changes in autonomic activity during 18 rTMS sessions in the same children with ASD. Our hypothesis was that children with ASD would show improved HRV measures (decreased overall HR indexed by longer R-R intervals, increased STDRR, higher pNN50 index, increased HF power, decreased LF power, decreased LF/HF ratio, increased pNN50) and lower SCL measures. Our results showed that, except for a reduction in LF power and pNN50, all dependent HRV variables changed in the predicted way, as indexed by statistically significant liner regression coefficients over TMS sessions and statistically significant pre- *vs*. post-TMS changes (first *vs*. last TMS session). The LF power decrease showed a trend towards decrease but it did not reach significance level.

Time-domain HRV results showed that the most significant changes from TMS treatment were an increase in R-R cardiointerval length and a higher standard deviation of R-R intervals. Frequency-domain HRV results showed increase of HF power in HRV, and decreased LF/HF ratio. Electrodermal activity also showed a decrease in the form of lower tonic SCL. The increased standard deviation in cardiointervals along with higher power of HF of HRV and decreased LF/HF ratio are promising because this suggests more prominent parasympathetic activity and more flexibility in HR overall. Significant change was also observed in mean R-R interval lengths, which means a lower HR. Outcomes within the frequency-domain of HRV showed increased HF component of HRV, which is also of importance as it suggests enhancement of the parasympathetic tone. As we did not observe a statistical change in the LF component, it can be inferred that restoration of autonomic balance was achieved mainly through an increased HF component of HRV, which correlates to parasympathetic (vagus) cardiac neural control. However, while the change in the LF component was not significant, we did observe a decrease in SCL over the 18 sessions. This result suggests a withdrawal of sympathetic tone as SCL is controlled by sympathetic inputs. It should be noted that cardiac sympathetic influences are predominantly mediated through beta-adrenergic drives, while peripheral sympathetic control of sweat glands is exerted through alpha-adrenergic drives.

The question remains as to how does prefrontal rTMS affect autonomic functions? Only a few papers have looked at the effects of rTMS on the autonomic system, despite the fact that many frontal cortical areas are directly implicated in ANS control (Filippi et al., 2000; Czéh et al., 2002). It has been reported (Ben-Shachar et al., 1997) that there might be neurohumoral changes after treatment with rTMS. A hypothesis was also proposed suggesting that anxiolytic effects of rTMS may act through normalization of the hypothalamic-pituitary-adrenocortical (HPA) axis (Holsboer, 2000). Chronic rTMS-induced changes in stress-related corticotropin and corticosterone levels have been found in animal models (Keck et al., 2000; Hedges et al., 2002) providing support for the suggestion that rTMS, directed at the prefrontal lobe, may attenuate the activity of the HPA system.

Low frequency rTMS can influence autonomic balance when using HRV (Yoshida et al., 2001). Udupa et al. (2007) reported that HRV measures indicated that rTMS produced a significant reduction in the cardiac sympathetic/vagal ratio, suggesting improvements in the sympatho-vagal cardiac balance. Lower post-TMS sympathetic activity was reported in one additional study (Jenkins et al., 2002). It is possible that rTMS effects are mediated through fronto-limbic connections. The limbic system is a complex network of structures central to anxiety and mood regulation (Mayberg, 2003; Seminowicz et al., 2004). Originally rTMS was investigated as a potential antidepressant therapeutic device under the assumption that magnetic stimulation of the prefrontal cortex (PFC) would engage the connected limbic regions involved in mood and anxiety regulation (George et al., 1999). The hypothesis is consistent with the PFC rTMS modulating the function of fronto-limbic circuits.

Another important question is how TMS affects cortical E/I balance. Several studies have outlined a disruption in the ratio between cortical excitation and inhibition in ASD (Casanova et al., 2002; Rubenstein and Merzenich, 2003; Casanova, 2006; Yizhar et al., 2011). One possible explanation for an increase in cortical excitation to inhibition in ASD is the recent finding of abnormalities in cortical minicolumns (Casanova et al., 2002; Casanova, 2005). Double-bouquet cells in the peripheral neuropil space of minicolumns impose a strong vertically directed stream of inhibition (Mountcastle, 2003) surrounding the minicolumnar core. In ASD our preliminary studies indicate that cortical minicolumns are reduced in size and increased in number, especially within the prefrontal cortex (Casanova et al., 2002, 2006; Casanova, 2005, 2006). Disturbances in the ratio of cortical excitation to inhibition may lead to an increase in cortical “noise” that may influence functional cortical connectivity and may hinder the “binding” of associated cortical areas. It has been proposed that the effect of “slow” rTMS arises from increases in the activation of inhibitory circuits. We theorize that contrary to other inhibitory cells (i.e., basket and chandelier), whose projections keep no constant relation to the surface of the cortex, the geometrically exact orientation of double-bouquet cells and their location at the periphery of the minicolumn makes them an appropriate candidate for induction by a magnetic field applied parallel to cortex. Over a course of treatment rTMS may selectively lower the ratio of cortical excitation to cortical inhibition. Low frequency rTMS over DLPFC may therefore lead to improvement in frontal functions, including fronto-limbic function. We have already reported positive effects of rTMS in autism as expressed in improved ERP and evoked and induced gamma frequency oscillations during performance on visual oddball task (Sokhadze et al., 2009a,b, 2010a,b, 2012a; Baruth et al., 2010a,b; Casanova et al., 2012). The main finding was improved target discrimination and attenuated responses to non-target items, indicative of better differentiation of targets vs. non-targets and improved early stage filtering of task-irrelevant stimuli.

By convention, rTMS in 0.3–1 Hz frequency range is referred to as “slow,” whereas “fast” rTMS refers to stimulation greater than 5 Hz. Hoffman and Cavus (2002) in their review of slow rTMS studies proposed long-term depression and long-term depotentiation as models for understanding the mechanism of slow rTMS. Neocortical long-term depression and changes in the cortical excitability induced by slow rTMS appear to accumulate in an additive fashion as the number of stimulations is increased over many days. Studies of both slow rTMS and long-term depression suggest additive efficacy when higher numbers of spaced, daily stimulations are administered. The reversal, or depotentiation, of previously enhanced synaptic transmission due to long-term potentiation may be the most relevant model for slow rTMS when used as a therapeutic tool. Our study used relatively high number of slow rTMS sessions (18 sessions on weekly rate). The mechanism of low-frequency TMS involves increasing inhibition of the stimulated cortex. For this study the stimulated region was the DLPFC, which is linked to the tonic inhibitory control of the ANS activity. The findings of our study indicate that TMS applied to the DLPFC was successful in the positive modulation of the autonomic balance in ASD through activation of the parasympathetic tone and withdrawal of sympathetic tone.

Some potential implications of TMS based neuromodulation could be considered in the context of other stimulation approaches and comorbidities proper to ASD. Excessive sympathetic arousal is often associated with anxiety. For children with ASD, especially during adolescence, anxiety is one of the most common presenting problems in clinical settings. Several research groups have reported that over 55% of sampled children with ASD meet criteria for at least one anxiety disorder (de Bruin et al., 2007; McPheeters et al., 2011). Anxiety disorder, as in social phobia, may be related to reduced functional connectivity between the frontal lobes and the limbic system (Hahn et al., 2011). Development of new neuromodulation methods aimed at regulating the effect of the frontal lobe on autonomic functions may thus provide a potential therapeutic intervention in ASD. Some potential implications of TMS based neuromodulation in autism could be considered also in the context of other stimulation approaches, for instance Vagal Nerve Stimulation (VNS) applications in ASD (Levy et al., 2010). Future studies of adequate sample size and sham controls are needed to explore the use of rTMS as a novel treatment for improving autonomic balance in ASD.

## Conflict of interest statement

The authors declare that the research was conducted in the absence of any commercial or financial relationships that could be construed as a potential conflict of interest.
